# Two Distinct Pathways to Development of Squamous Cell Carcinoma of the Vulva

**DOI:** 10.1155/2011/951250

**Published:** 2010-09-28

**Authors:** Yutaka Ueda, Takayuki Enomoto, Toshihiro Kimura, Kiyoshi Yoshino, Masami Fujita, Tadashi Kimura

**Affiliations:** Department of Obstetrics and Gynecology, Osaka University Graduate School of Medicine, 2-2, Yamadaoka, Suita, Osaka, 565-0871, Japan

## Abstract

Squamous cell carcinoma (SCC) accounts for approximately 95% of the malignant tumors of the vaginal vulva and is mostly found in elderly women. The future numbers of patients with vulvar SCC is expected to rise, mainly because of the proportional increase in the average age of the general population. Two different pathways for vulvar SCC have been put forth. The first pathway is triggered by infection with a high-risk-type Human Papillomavirus (HPV). Integration of the HPV DNA into the host genome leads to the development of a typical vulvar intraepithelial neoplasia (VIN), accompanied with overexpression of p14^ARF^ and p16^INK4A^. This lesion subsequently forms a warty- or basaloid-type SCC. The HPV vaccine is a promising new tool for prevention of this HPV related SCC of the vulva. The second pathway is HPV-independent. Keratinizing SCC develops within a background of lichen sclerosus (LS) through a differentiated VIN. It has a different set of genetic alterations than those in the first pathway, including p53 mutations, allelic imbalances (AI), and microsatellite instability (MSI). Further clinical and basic research is still required to understand and prevent vulvar SCC. *Capsule*. Two pathway for pathogenesis of squamous cell carcinoma of the value are reviewed.

## 1. Introduction

Squamous cell carcinoma (SCC) accounts for only 5% of the malignant tumors of the female genital tract, but it represents 95% of vaginal vulvar tumors [1]. The incidence of malignant vulvar tumors in the United States is 1.5 per 100,000 women per year, and this incidence increases with age. The average age at diagnosis is in the 7th to 8th decades of life, with a future rise in absolute numbers of vulvar SCC expected, mainly due to the proportional increase in the average age of the general population [2].

Two different types of vulvar SCC have been delineated, each with their own precursors. The first type is associated with an infection with one of the high-risk types of Human Papillomaviruses (HPV), and it primarily affects younger women. The other type is associated with a lichen sclerosus (LS) condition, and it occurs predominantly in elderly patients independent of any HPV infection [2]. Although the pathogenesis of vulvar SCC has been investigated, it has not been documented nearly as well as the pathogenesis of the more common cervical SCC. 

We herein give an overview, focusing on the molecular events of these two distinct HPV-associated and independent pathways for the development of vulvar SCC.

## 2. Clinical and Pathological Features of SCC and Its Precursors

### 2.1. SCC

Vulvar SCC accounts for 90% of vulvar cancers and 5% of gynecological cancers. Patients usually present with a vulvar mass, which may be pruritic or painful or be associated with bleeding, and, occasionally, with a groin mass. Clinical factors that have adverse prognostic significance include increased stage, older age, smoking, and fixed or ulcerated groin nodes [3].

**Figure 1:**
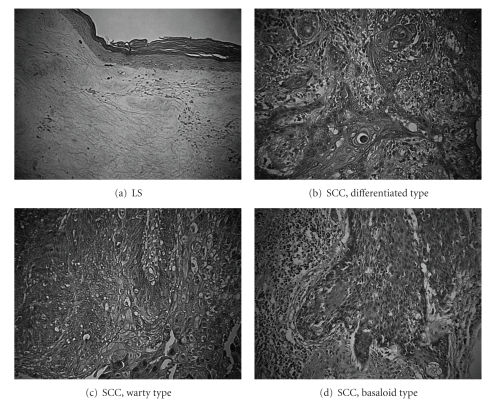
Examples of hematoxylin and eosin staining of vulvar lesions (×200) type.

The three main histological subtypes of vulvar SCC are: warty, basaloid, and keratinizing (Table 1 and Figure 1). The predominant type, keratinizing, accounts for 65%–80% of vulvar SCCs; the basaloid and warty types of SCC account for the remaining 20%–35% [3]. The keratinizing type usually occurs in postmenopausal women; the warty/basaloid types tend to occur more often in premenopausal or perimenopausal women. The keratinizing type is usually formed by well, or moderately differentiated cells with an absence of koilocytosis. There are usually one or more adjacent epithelial lesions, including LS, squamous cell hyperplasia (SCH), and differentiated vulvar intraepithelial neoplasia (VIN) [3], which will each be described further in the following sections.

The warty or basaloid types of SCC often accompany a normal-type VIN. The basaloid type typically grows in bands, sheets, or nests within a desmoplastic stroma, and focal cytoplasmic maturation and keratinization may be observed. The warty type exhibits invasion as bulbous or irregular jagged nests, often with prominent keratinization. The koilocytotic tumor cells have pleomorphic to bizarre, often multiple, nuclei with irregular contours that vary from hyperchromatic and shrunken to those with clumped or smudged chromatin [3]. 

Other histological subtypes include verrucous carcinoma, giant cell carcinoma, and acantholytic squamous cell carcinoma. Verrucous carcinoma is highly differentiated squamous carcinoma that has a verrucous pattern and invades with a pushing border in the form of bulbous pegs of neoplastic cells. Squamous cell carcinoma with tumor giant cells is a variant of SCC characterized by multinucleated tumor giant cells, large nuclei with prominent nucleoli, and prominent eosinophilic cytoplasm. This variant is relatively rare and is associated with a poor prognosis. The acantholytic squamous cell carcinoma forms rounded spaces, or pseudoacini, lined with a single layer of squamous cells. Dyskeratotic and acantholytic cells are sometimes present in the central lumen [4].

### 2.2. VIN

Various terms have been used to define the precursors of vulvar SCC. Bowen first reported on these squamous intraepithelial lesions in 1912, and they are now commonly referred to as Bowen's disease; since then, a myriad of clinical and histopathological terms have been employed to describe these vulvar precancerous lesions [5]. The International Society for the Study of Vulvar Disease (ISSVD) simplified the terminology for carcinoma in situ and vulvar atypia in 1976; in 1986 they adopted the single term of VIN and a 3-grade VIN system based on the terminology from cervical intraepithelial neoplasia (CIN) [5]. In VIN 1, maturation was present in the upper two-thirds of the epithelium. In VIN 2, the dysplasia involves the lower two-thirds of the epithelium, and in VIN 3, the dysplasia extends into the upper third [5]. The terms of warty, basaloid, and differentiated (simplex) are used in the same way as for cervical SCC. 

The most recent classifications are shown in Table 2. The World Health Organization (WHO) classifies VIN according to the 3-grade system for both the warty/basaloid types and the simplex type [7]. In 2004, ISSVD modified their VIN terminology and suggested a 2-tier classification: VIN usual type and VIN differentiated type. Moreover, they decided to abolish the term VIN 1. The term of VIN is now applied only to the histologically high-grade squamous lesions that were the former VIN 2 and VIN 3 or differentiated VIN [5]. This revision was made based on the observation that there was neither evidence that the morphologic spectrums of VIN 1, 2, and 3 reflect a biologic continuum nor that VIN 1 was a cancer precursor [2]. In 2005, Medeiros et al. proposed a Bethesda-like grading system of low-grade vulvar intraepithelial lesions (Low-grade VILs) and high-grade vulvar intraepithelial lesions (High-grade VILs) [6]. Low-grade VILs correspond to lesions associated with low-risk HPV infections. Condyloma acuminatum and discrete raised lesions with minimal atypia and lacking the features of dermatosis (VIN 1) were categorized into low-grade VIL [6]. 

A systematic review of the progression rate from VIN 3 to invasive SCC, after various clinical treatments, was reported to be 3.3% [8]. Nine percent of 88 untreated patients progressed to SCC during 12 to 96 months. Complete regression of usual VIN 3 lesions were observed in 1.2% of 3322 patients, mostly during the first 10 months after diagnosis, 41% of which remission was related to pregnancy. Another study demonstrated that the overall percentage of differentiated VIN lesions with subsequent diagnosis of SCC was 32.8%, and that of usual type VIN was 5.7% [9]. The median time for progression from usual type VIN to SCC was 41.4 months whereas that from differentiated VIN to SCC was significantly shorter: 22.8 months (*P* = .005). Another study demonstrated that the mean time from the incidence of HPV infection to the development of VIN 1–3 was 18.5 months (95% confidence interval, 13.4–23.6) [10]. 

The typical presentation of usual VIN is a pruritic, burning, or asymptomatic, white, red, or pigmented lesion. The incidence of this form of VIN has almost doubled over the past several decades, with a significant increase in younger women. The lesions of differentiated VIN usually range from 0.5 to 3.5 cm, appearing as single or multiple gray-white areas with a rough surface or ill-defined white plaques or nodules. The lesions usually occur in postmenopausal women [3].

### 2.3. LS

LS runs a relapsing and remitting course and presenting symptoms include pruritus, soreness, burning, and irritation. Typically, the lesions are white plaques and papules, often with areas of erythema hyperkeratosis, pallor, and ulcer [2].

The histological features of lichen sclerosus (LS) include a thinned epidermis with loss of normal rete pegs, basal layer vacuolar changes and a paucity of melanocytes, and, moreover, a wide band of homogeneous collagen below the dermatoepidermal junction and a band-like lymphocytic infiltrate below the homogenized area are present (Figure 1). The dermis often shows variable degrees of edema [2]. 

LS most commonly affects the anogenital area (85% to 98%), with extragenital lesions occurring in 15% to 20% of the patients [11]. LS occurs at all ages; however, it has a bimodal peak of incidence in prepubertal girls and postmenopausal women [2]. According to a previous study, 1 out of 30 elderly women suffer from LS [12]. As association of LS with autoimmune disorders has been demonstrated. According to previous studies, around 30% of the patients have active autoimmune disease and autoantibodies were detected in about half of the serum of the LS patients [2, 13–16]. LS is considered to occur at sites of injured skin in women with the susceptible immunophenotype who scratch the area because of genital irritation [2]. 

The risk of development of vulvar SCC in women with LS was shown to be 4% to 5% [11]. A previous review study also estimated a 4.5% frequency of SCC arising in LS with an interval of approximately 10 years (1.67 to 12.5 years) after diagnosis of LS without SCC [17].

## 3. Mechanisms of Carcinogenesis

### 3.1. HPV-Related Carcinogenesis

**Figure 2:**
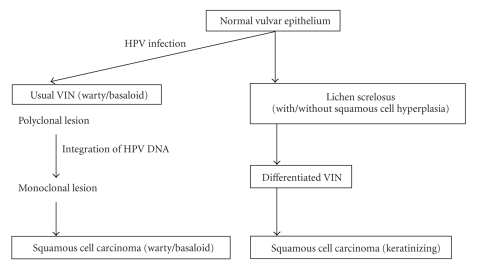
Pathogenesis of squamous cell carcinoma of the vulva. (Distinct pathways for carcinogenesis of keratinizing and warty/basaloid types of vulvar SCC from normal epithelium through precursor lesions are demonstrated.)

Two distinct pathways, HPV related and HPV independent, were proposed for vulvar carcinogenesis (Figure 2). Warty/basaloid type SCC develops through usual (warty/basaloid) type VIN triggered by infection with high-risk type HPVs, predominantly HPV−16 and −18 [2]. Usual type VIN lesions are observed adjacent to greater than 10%–67% of the vulvar SCC lesions [18]. A previous study showed that 69% to 100% of warty/basaloid type SCC were positive for high-risk type HPVs [19]. High-risk type HPVs were detected in 84% of 45 usual VIN cases [20]. Eighty-seven percent (13 of 15) of the usual high-risk type HPV-positive VIN lesions were shown to express both p14^ARF^ (a cell-cycle regulator which mediates p53 activation) and p16^INK4A^ (a cyclin-dependent kinase inhibitor) [21]. Hoevenaars showed that all 38 usual VIN lesions exhibited positive p16^INK4A^ immunohistochemical staining, and that in all these cases a high MIB1 index was observed. No expression of p53 and p16^INK4A^ was detected in normal epithelium of the vulva [20]. However, so far little has been found concerning the mechanism of enhanced expression of p14^ARF^ and p16^INK4A^ in the carcinogenesis of vulvar SCC. 

The HPV viral gene products E6 and E7 interact with host cell p53 and Rb proteins, resulting in p53 dysfunction and inactivation of Rb, respectively. In cervical carcinogenesis triggered by high-risk type HPV infection, E7 inhibits Rb, resulting in the release of active host E2F-1, which positively regulates host p14^ARF^. E6 inhibits p53 function by binding with E6-AP ubiquitin ligase, and leads to p14^ARF^ upregulation via p53 degradation by negative feedback mechanism [22, 23]. Functional inactivation of Rb by E7 protein also leads to p16^INK4A^ overexpression. Taken together, p14^ARF^ and p16^INK4A^ were over-expressed as a consequence of HPV E6 and E7 expression in cervical carcinomas. 

In carcinogenesis of HPV-related SCC, similar mechanisms to cervical carcinogenesis seem to play an important role. Degradation and inactivation of the tumor suppressor genes p53 and Rb leads to absence of cell-cycle arrest and hyperproliferation of tumor cells. Frequent detection of over-expression of p14^ARF^ and p16^INK4A^ in VIN suggests that degradation and inactivation of p53 and Rb are early events in carcinogenesis of HPV-related SCC of the vulva.

#### 3.1.1. Integration of High-Risk Type HPV DNA

In uterine cervical carcinogenesis, integration of the high-risk type HPV DNA into the host genome was demonstrated to be an initial step for monoclonal expansion of dysplastic cells [24]. In the process of the integration, some parts of the E2 open reading frame (ORF), which encode a 48-kd phosphorylated protein involved in the regulation of viral DNA transcription and replication, are usually disrupted or deleted from HPV genome, causing up-regulation of the oncogenic E6 and E7 genes [25]. HPV integration sites were demonstrated to be semirandomly distributed over the whole genome, with a clear predilection for genomic fragile sites, but there was no evidence for targeted disruption or functional alteration of critical cellular genes by the integrated viral sequences. The main function of HPV integration is considered to be for the stabilization of viral oncogene transcription [26, 27]. 

Similar mechanisms to those for cervical cancer seem to play an important role during the development of HPV-related vulvar SCC triggered by high-risk type HPV infection. Integration of HPV-16 DNA, with deletion of the E2 ORF in both the SCC portion and its adjacent VIN 3 lesions, which were all implied to be formed from a single cell of origin through monoclonal expansion, was first demonstrated in a case of vulvar SCC by Ueda et al. [35]. Monoclonal composition was also demonstrated in 3 of 7 cases of VIN 1/2, and 12 of 13 VIN 3 cases in the study. Later, additional supporting studies have also shown VIN 3 cases associated with infection of HPV in an integrated form [28, 29].

#### 3.1.2. HPV Vaccine

The HPV vaccine is a tremendously promising new tool for the prevention of HPV-related SCC of the vulva, as it has already been for the cervix. The FUTURE I study has demonstrated that a prophylactic quadrivalent HPV-(6/11/16/18) L1 VLP vaccine significantly reduced the incidence of HPV-associated anogenital diseases in young women [30]. The prophylactic efficacy in the study was 100% for vulvar condyloma, VIN 1, and VIN 2/3 in the per-protocol population. Other studies also demonstrated that the prophylactic quadrivalent HPV vaccine completely protected VIN 2/3 [31, 32]. 

Interestingly, a series of 3-4 vaccinations against a synthetic long-peptide of the HPV-16 oncoproteins E6 and E7 was shown to be therapeutically effective for HPV-16-positive VIN 3 patients [33]. At 3 months after the last vaccination, 5 (25%) of 20 patients had complete remission of the lesion, and HPV-16 was no longer detected in 4 cases (20%). At 12 month of followup, 9 (47%) of 19 patients had a complete response with tolerable adverse effects. The patients who had a complete response at 3 months demonstrated a significantly stronger interferon-*γ*-associated proliferative CD4+ T-cell response and a broader response of CD8+ interferon-*γ* T-cells. A phase II clinical trial of the topical immune-modulator imiquimod, followed by therapeutic HPV-16 vaccine using a fusion protein of HPV-16 E6E7L2 on 19 cases with VIN 2/3, demonstrated that complete regression of VIN 2/3 lesions was observed in 63% of the cases (12 of 19) [34].

### 3.2. HPV-Independent Carcinogenesis

The majority of vulvar SCC are considered to occur in elderly women through differentiated VIN in a background of LS [2]. High-risk type HPVs were detected in none of 75 differentiated VIN cases [20]. They also showed that all 75 differentiated VIN lesions exhibited negative p16 immunohistochemical staining, and a low MIB1 index was observed in 96% (72 of 75 cases) of the cases [20]. No relationship between HPV infection and LS was found in these women [2, 21]. These results strongly suggest that an HPV-independent pathway exists for carcinogenesis of vulvar SCC from LS through differentiated VIN; however, the mechanism of HPV-independent carcinogenesis has not yet been fully elucidated. 

We have previously demonstrated that 2 of 6 LS lesions exhibited monoclonality, implying that certain important molecular alterations might occur in some LS lesions well before histologically apparent malignant transformation to differentiated VIN or keratinizing SCC occurs [35]. Rolfe et al. showed that 10 of 12 LS-associated SCCs exhibited a p53 mutation, and in 7 of those 10 cases LS lesions exhibited the p53 mutation at the same codon as in the SCC lesions, suggesting that a p53 mutation is possibly involved early in the HPV-independent pathway of vulvar carcinogenesis [36]. Somatic mutation of PTEN was also demonstrated in some cases of vulvar SCC and VIN, suggesting that PTEN mutation possibly played a role early in the carcinogenesis of vulvar SCC [37]. Pinto et al. found that an allelic imbalance (AI) was present in 67%, 53%, and 43% of usual type VIN, differentiated VIN and LSs, respectively, and that microsatellite instability (MSI) was detected in 3 (20%) of 15 differentiated VIN, and 2 (12%) of 17 LS, but none of usual type VIN, implying that these molecular alterations are also possibly early events in vulvar carcinogenesis, and that MSI may play a critical role for malignant potential of LS [38]. 

A recent study demonstrated more frequent hypermethylation of RASSF2A, MGMT, and TSP-1 genes in SCC associated with LS than in SCC not associated with LS, suggesting a possible role of these genes in HPV-independent carcinogenesis [39]. 

A fraction of squamous cell hyperplasia (SCH) lesions were shown to be monoclonal in composition [35] and p53 mutation, AI, and MSI were observed in 22%, 50% and 20%, respectively, of SCH cases [36, 38]. SCH with atypia might be a precursor of SCC; however, SCH without atypia is, currently, not regarded as a direct precursor of SCC. Relationship between SCH and keratinizing SCC is still to be determined [2, 38].

## 4. Conclusions

Two distinct pathways leading to vulvar SCC have been suggested. One is a pathway primarily linked to infection with high-risk types of HPV; the other is an HPV-independent scenario. Mechanisms similar to those that drive cervical carcinogenesis possibly play an important role in HPV-related carcinogenesis of vulvar SCC. HPV prophylactic and therapeutic vaccines are both promising to prevent HPV infection and prevent development of warty/basaloid type SCC from its precursor, the usual type VIN. On the other hand, keratinizing type vulvar SCC, which by far represents the majority of vulvar SCC, occurs independently from HPV infection in a background of LS. The mechanism of carcinogenic progression forward from LS in this second pathway has not fully delineated, and it is not yet clear whether medical treatments of LS prevent malignant transformation. Further clinical and basic research into these important areas is still required.

## Figures and Tables

**Table 1:** Characteristics of two types of squamous cell carcinoma of the vulva. (Characteristics of the warty/basaloid type and the keratinizing type of SCC of the vulva are shown).

	warty or basaloid type	keratinizing type
Frequency	20%–35%	65%–80%
Age	Younger	Older
	55 (35–65)	77 (55–85)
Precursor	warty or basaloid VIN	Lichen sclerosus differentiated VIN
Molecular characteristics	HPV integration	p53 mutation
	p14^ARF^·p16^INK4a^ overexpression	Microsatellite instability
Prognosis	better	worse

**Table 2:** Classifications of vulvar intraepithelial neoplasia. (WHO, ISSVD, and Bethesda-like classifications are shown.)

	WHO (2003)
	VIN 1,2,3 (warty type/basaloid type)
	VIN 1,2,3 (simplex type)
	ISSVD (2004)*
	VIN, usual type (VIN 2,3)
	(a) warty type
	(b) basaloid type
	(c) mixed type
	VIN 3, differentiated type (VIN 3)
	Bethesda-like system [6]
	Low-grade VIL (condyloma NIN 1)
	High-grade VIL (VIN 2/VIN 3)

*VIN1: abolished terminology.

## Abbreviations

AI: Allelic imbalance
CIN: Cervical intraepithelial neoplasia
HPV: Human Papillomavirus
ISSVD: The International Society for the Study of Vulvar Disease
LS: Lichen sclerosus
MSI: Microsatellite instability
SCC: Squamous cell carcinoma
SCH: Squamous cell hyperplasia
VIL: Vulvar intraepithelial lesions
VIN: Vulvar intraepithelial neoplasia
WHO: World Health Organisation.

## References

[1] Kurman RJ, Norris HJ, Wilkinson E (1998). Tumors of the cervix, vagina, and vulva. *Atlas of Tumor Pathology, Third Series, Fascicle 4*.

[2] van de Nieuwenhof HP, van der Avoort IAM, de Hullu JA (2008). Review of squamous premalignant vulvar lesions. *Critical Reviews in Oncology/Hematology*.

[3] Clement PB, Young RH (2000). *Atlas of Gynecologic Surgical Pathology*.

[4] Kurman RJ (1994). *Blaunstein's Pathology of the Female Genital Tract*.

[5] Preti M, Van Seters M, Sideri M, Van Beurden M (2005). Squamous vulvar intraepithelial neoplasia. *Clinical Obstetrics and Gynecology*.

[6] Medeiros F, Nascimento AF, Crum CP (2005). Early vulvar squamous neoplasia: advances in classification, diagnosis, and differential diagnosis. *Advances in Anatomic Pathology*.

[7] Wilkinson EJ, Teixeira MR (2003). Epithelial tumours, squamous tumours. *World Health Organisation Classification of Tumours: Pathology and Genetics: Tumours of the Breast and Female Genital Organs*.

[8] Van Seters M, Van Beurden M, De Craen AJM (2005). Is the assumed natural history of vulvar intraepithelial neoplasia III based on enough evidence? A systematic review of 3322 published patients. *Gynecologic Oncology*.

[9] van de Nieuwenhof HP, Massuger LFAG, van der Avoort IAM (2009). Vulvar squamous cell carcinoma development after diagnosis of VIN increases with age. *European Journal of Cancer*.

[10] Garland SM, Insinga RP, Sings HL, Haupt RM, Joura EA (2009). Human papillomavirus infections and vulvar disease development. *Cancer Epidemiology Biomarkers and Prevention*.

[11] Powell JJ, Wojnamwska F (1999). Lichen sclerosus. *The Lancet*.

[12] Leibovitz A, Kaplun V, Saposhnicov N, Habot B (2000). Vulvovaginal examinations in elderly nursing home women residents. *Archives of Gerontology and Geriatrics*.

[13] Goolamali SK, Barnes EW, Irvine WJ, Shuster S (1974). Organ specific antibodies in patients with lichen sclerosus. *British Medical Journal*.

[14] Meyrick Thomas RH, Ridley CM, McGibbon DH, Black MM (1988). Lichen sclerosus et atrophicus and autoimmunity—a study of 350 women. *British Journal of Dermatology*.

[15] Harrington CI, Dunsmore IR (1981). An investigation into the incidence of auto-immune disorders in patients with lichen sclerosus and atrophicus. *British Journal of Dermatology*.

[16] Oyama N, Chan I, Neill SM (2003). Autoantibodies to extracellular matrix protein 1 in lichen sclerosus. *The Lancet*.

[17] Carlson JA, Ambros R, Malfetano J (1998). Vulvar lichen sclerosus and squamous cell carcinoma: a cohort, case control, and investigational study with historical perspective; implications for chronic inflammation and sclerosis in the development of neoplasia. *Human Pathology*.

[18] Maclean AB (2006). Vulvar cancer: prevention and screening. *Best Practice and Research: Clinical Obstetrics and Gynaecology*.

[19] Riethdorf S, Neffen EF, Cviko A, Löning T, Crum CP, Riethdorf L (2004). p16INK4A expression as biomarker for HPV 16-related vulvar neoplasias. *Human Pathology*.

[20] Hoevenaars BM, Van Der Avoort IAM, De Wilde PCM (2008). A panel of p16INK4A, MIB1 and p53 proteins can distinguish between the 2 pathways leading to vulvar squamous cell carcinoma. *International Journal of Cancer*.

[21] van der Avoort IAM, Shirango H, Hoevenaars BM (2006). Vulvar squamous cell carcinoma is a multifactorial disease following two separate and independent pathways. *International Journal of Gynecological Pathology*.

[22] Sano T, Masuda N, Oyama T, Nakajima T (2002). Overexpression of p16 and p14ARF is associated with human papillomavirus infection in cervical squamous cell carcinoma and dysplasia. *Pathology International*.

[23] Kanao H, Enomoto T, Ueda Y (2004). Correlation between p14ARF/p16INK4A expression and HPV infection in uterine cervical cancer. *Cancer Letters*.

[24] Ueda Y, Enomoto T, Miyatake T (2003). Monoclonal expansion with integration of high-risk type human papillomaviruses is an initial step for cervical carcinogenesis: association of clonal status and human papillomavirus infection with clinical outcome in cervical intraepithelial neoplasia. *Laboratory Investigation*.

[25] Kalantari M, Karlsen F, Kristensen G, Holm R, Hagmar B, Johansson B (1998). Disruption of the E1 and E2 reading frames of HPV 16 in cervical carcinoma is associated with poor prognosis. *International Journal of Gynecological Pathology*.

[26] Wentzensen N, Vinokurova S, Von Knebel Doeberitz M (2004). Systematic review of genomic integration sites of human papillomavirus genomes in epithelial dysplasia and invasive cancer of the female lower genital tract. *Cancer Research*.

[27] Ziegert C, Wentzensen N, Vinokurova S (2003). A comprehensive analysis of HPV integration loci in anogenital lesions combining transcript and genome-based amplification techniques. *Oncogene*.

[28] Hillemanns P, Wang X (2006). Integration of HPV-16 and HPV-18 DNA in vulvar intraepithelial neoplasia. *Gynecologic Oncology*.

[29] Nakanishi G, Fujii K, Asagoe K, Tanaka T, Iwatsuki K (2009). Human papillomavirus genome integration in multifocal vulvar Bowen's disease and squamous cell carcinoma. *Clinical and Experimental Dermatology*.

[30] Garland SM, Hernandez-Avila M, Wheeler CM (2007). Females united to unilaterally reduce endo/ectocervical disease (FUTURE) I investigators. quadrivalent vaccine against human papillomavirus to prevent anogenital diseases. *The New England Journal of Medicine*.

[31] Kjaer SK, Sigurdsson K, Iversen O-E (2009). A pooled analysis of continued prophylactic efficacy of quadrivalent human papillomavirus (types 6/11/16/18) vaccine against high-grade cervical and external genital lesions. *Cancer Prevention Research*.

[32] Majewski S, Bosch F, Dillner J (2009). The impact of a quadrivalent human papillomavirus (types 6, 11, 16, 18) virus-like particle vaccine in European women aged 16 to 24. *Journal of the European Academy of Dermatology and Venereology*.

[33] Kenter GG, Welters MJP, Valentijn ARPM (2009). Vaccination against HPV-16 oncoproteins for vulvar intraepithelial neoplasia. *The New England Journal of Medicine*.

[34] Daayana S, Elkord E, Winters U (2010). Phase II trial of imiquimod and HPV therapeutic vaccination in patients with vulval intraepithelial neoplasia. *British Journal of Cancer*.

[35] Ueda Y, Enomoto T, Miyatake T (2004). Analysis of clonality and HPV infection in benign, hyperplastic, premalignant, and malignant lesions of the vulvar mucosa. *American Journal of Clinical Pathology*.

[36] Rolfe KJ, MacLean AB, Crow JC, Benjamin E, Reid WMN, Perrett CW (2003). TP53 mutations in vulval lichen sclerosus adjacent to squamous cell carcinoma of the vulva. *British Journal of Cancer*.

[37] Holway AH, Rieger-Christ KM, Miner WR (2000). Somatic mutation of PTEN in vulvar cancer. *Clinical Cancer Research*.

[38] Pinto AP, Lin M-C, Sheets EE, Muto MG, Sun D, Crum CP (2000). Allelic imbalance in lichen sclerosus, hyperplasia, and intraepithelial neoplasia of the vulva. *Gynecologic Oncology*.

[39] Guerrero D, Guarch R, Ojer A Differential hypermethylation of genes in vulvar cancer and lichen sclerosus coexisting or not with vulvar cancer.

